# Role of Oxidative Stress in Hepatocarcinogenesis Induced by Hepatitis C Virus

**DOI:** 10.3390/ijms131115271

**Published:** 2012-11-19

**Authors:** Kyoko Tsukiyama-Kohara

**Affiliations:** Department of Animal Hygiene, Transboundary Animal Diseases Center, Joint Faculty of Veterinary Medicine Kagoshima University, 1-21-24 Korimoto, Kagoshima 890-0065, Japan; E-Mail: kkohara@agri.kagoshima-u.ac.jp; Tel./Fax: +81-99-285-3589

**Keywords:** hepatitis C virus, reactive oxygen species, 3β-hydroxysterol Δ24-reductase

## Abstract

Hepatitis C virus (HCV) easily establishes chronic hepatitis, cirrhosis, and hepatocellular carcinoma (HCC). During the progression of HCV infections, reactive oxygen species (ROS) are generated, and these ROS then induce significant DNA damage. The role of ROS in the pathogenesis of HCV infection is still not fully understood. Recently, we found that HCV induced the expression of 3β-hydroxysterol Δ24-reductase (DHCR24). We also found that a HCV responsive region is present in the 5′-flanking genomic promoter region of DHCR24 and the HCV responsive region was characterized as (−167/−140). Moreover, the transcription factor Sp1 was found to bind to this region in response to oxidative stress under the regulation of ataxia telangiectasia mutated (ATM) kinase. Overexpression of DHCR24 impaired p53 activity by suppression of acetylation and increased interaction with MDM2. This impairment of p53 suppressed the hydrogen peroxide-induced apoptotic response in hepatocytes. Thus, a target of oxidative stress in HCV infection is DHCR24 through Sp1, which suppresses apoptotic responses and increases tumorigenicity.

## 1. Introduction

Hepatitis C virus (HCV) is a member of the *Flaviviridae* family of RNA viruses, and possesses a positive-strand RNA genome [1]. HCV mainly replicates in the cytoplasm, but frequently establishes chronic infections, leading to the development of chronic hepatitis, cirrhosis, and hepatocellular carcinoma (HCC) [2,3]. The estimated worldwide prevalence of HCV infections is 2.2%–3.0% [4], and chronic HCV infection is a major global public health concern. HCV does not possess canonical oncogenes and is unable to integrate into the host genome, but easily establishes chronic infections, resulting in HCC with high frequency. The exact mechanism by which this occurs is not fully understood; however, possible mediators of HCV pathogenesis are reactive oxygen species (ROS). During chronic hepatitis, the immune response induces the production of ROS [5] and nitric oxide (NO) [6]. Furthermore, HCV viral nucleocapsid protein, an HCV core protein, was shown to increase oxidative stress in the liver [7,8]. Moreover, HCV affects the steady-state levels of a mitochondrial protein chaperone known as prohibitin, leading to impaired function of the mitochondrial respiratory chain with the overproduction of ROS [9]. On the other hand, HCV compromises some of the antioxidant systems, including haeme oxygenase-1 [10] and NADH dehydrogenase quinone 1 [9], resulting in the provocation of oxidative stress in the liver during HCV infections. Thus, HCV infections not only induce ROS overproduction, but also hamper the antioxidant system in the liver. The induction of oxidative stress also results in the generation of deletions in mitochondrial and nuclear DNA, which are indicators of genetic damage. NO has been shown to induce oxidative DNA damage and inhibit DNA repair [11–13]. These nucleotide abnormalities may contribute to the development of HCC [14].

## 2. Survey of HCV-Positive HCC-Related Host Factors

To define the host factors involved in hepatocarcinogenesis during HCV persistent infections, we established a human hepatoblastoma-derived cell line (HepG2), which expresses the full-length HCV genome under the control of a Cre/*lox*P system (RzM6 cells [15]). Using colony-formation assays and nude mice tumor-formation assays, we found that passaging of HCV-expressing cells (RzM6-LC cells) increased their tumorigenicity. To identify which pathway was responsible for the increase in tumorigenicity in RzM6-LC cells, we raised monoclonal antibodies against the RzM6-LC cells and characterized them [16]. We found that one of these clones (2-152a) recognizes 3β-hydroxysterol Δ24-reductase (or dehydrocholesterol reductase 24; DHCR24). DHCR24 functions as an enzyme that catalyzes the conversion of desmosterol to cholesterol in the post-squalene cholesterol biosynthesis pathway [17,18]. The absence of DHCR24 leads to desmosterosis [19]. Furthermore, expression of DHCR24 is down-regulated in areas of the brain affected by Alzheimer’s disease [20]. DHCR24 is a multifunctional enzyme, which exerts resistance against oxidative stress and prevents apoptotic cell death when it is expressed at high levels [20–24]. Endogenous DHCR24/seladin-1 levels are up-regulated in response to acute oxidative stress [21,25,26], but the expression levels decline upon chronic exposure to oxidative stress [21,22]. DHCR24 is also reported to function as a hydrogen peroxide scavenger [24]. Thus, DHCR24 plays a crucial role in maintaining cellular physiology by regulating both cholesterol synthesis and cellular defence against oxidative stress, although the biological relevance of the hydrogen peroxide concentration (0.5–2 mM) used in some experiments requires future study.

## 3. HCV Induces DHCR24 Expression through Oxidative Stress

Since we observed up-regulation of DHCR24 expression in RzM6-LC cells, we decided to characterize the effects of HCV on DHCR24 expression [16,27]. Silencing of HCV by siRNA in RzM6-LC cells down-regulated the expression of DHCR24. By using chimeric mice with humanized liver [28], HCV infection induced the up-regulation of DHCR24 expression in human hepatocytes, whereas hepatitis B virus (HBV) infection had no significant effect on DHCR24 expression [16]. The regulation of DHCR24 expression was elicited at the transcriptional level. Therefore, we cloned the 5′-flanking region of the predicted genomic promoter region of *DHCR24* (~5 kb) and characterized the promoter activity by construction of promoter reporter plasmids [27]. We transfected each HCV protein (core, E1, E2, NS2, NS3/4A, NS4B, NS5A, and NS5B) or the full-genome HCV. The full-genome HCV induced significantly higher DHCR24 expression than other HCV viral proteins. The serial deletion mutants of the 5′-flanking region of *DHCR24* revealed that the minimum responsive element to the full-genome HCV was between −167 and −140 of the *DHCR24* gene. An electronic mobility shift assay (EMSA) identified that the specific binding factor to this element was the Sp1 transcription factor.

Transcription of *DHCR24* was induced by oxidative stress and impaired by the removal of the HCV minimum responsive element. Furthermore, the augmentation of *DHCR24* expression was impaired by treatment with a ROS scavenger, *N*-acetylcysteine. We then explored the role of the Sp1 transcription factor in the regulation of *DHCR24* expression. Phosphorylation of Sp1 at Ser101 was elevated under oxidative stress and increased by the presence of HCV. This phosphorylation of Sp1 was mediated through ataxia telangiectasia mutated (ATM) kinase [29,30]. Sustained phosphorylation of ATM and delayed de-phosphorylation of histone H2AX at Ser139 (γH2AX) were observed in HCV replicon cells [27,31], indicating that DNA repair was impaired in cells expressing or replicating HCV.

Previous studies revealed that expression of the HCV gene elevates the level of ROS via dysregulation of ER-mediated calcium homeostasis, which results in oxidative stress [32]. Also, the HCV core protein inhibits mitochondrial electron transport and increases ROS [33]. Recently, HCV infection is reported to increase ROS production through NADPH oxidase activity, especially elevated NADPH oxidase 4 (Nox4) [34]. The production of ROS can induce DHCR24 expression [27]. Thus, our results raised the possibility that DHCR24 plays a role in response to ROS generated as a consequence of HCV infection, thereby suppressing DNA repair and promoting tumorigenicity.

## 4. Overexpression of DHCR24 Results in Impairment of p53 Activity

**Figure 1:**
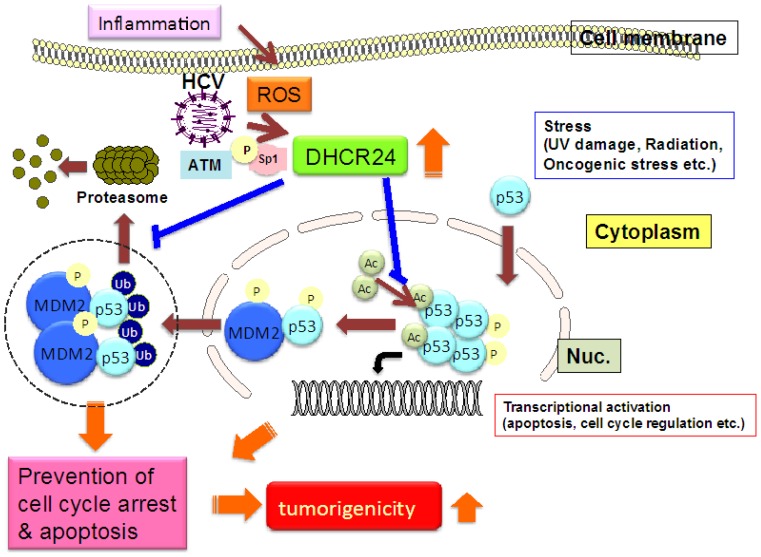
Elevation of tumorigenicity in HCV infected hepatocytes through increased oxidative stress and DHCR24.

HCV gene expression or infection persistently induces over-expression of DHCR24 [16,27] in its turn induces apoptotic resistance to oxidative stress (Figure 1).

HCV gene expression elevates the levels of ROS through dysregulation of ER-mediated calcium homeostasis. This increases the level of SP1 phosphorylation by ATM kinase, and results in the transcriptional activation of the *DHCR24* gene. The augmentation of DHCR24 by HCV suppresses p53 activity by blocking nuclear p53 acetylation and increasing the interaction between p53 and HDM2 (p53-specific E3 ligase) in the cytoplasm, which may be mediated by inhibition of p53 degradation. This impairment of p53 activity may result in apoptotic resistance and increased tumorigenicity.

To further examine this mechanism, we characterized the regulatory proteins involved in the oxidative stress-induced apoptotic response and found that p53 activity was impaired in response to hydrogen peroxide, which was clarified by a p21*^WAF1/CIP1^* promoter reporter assay. The post-translational modification of p53 after hydrogen peroxide treatment was characterized, and we found that the acetylation of p53 at Lys^373^ and Lys^382^ was impaired by the over-expression of DHCR24. The decreased level of p53 acetylation may impair p53 sequence-specific DNA-binding activity [35] and stability [36,37]. Moreover, interaction of p53 with its specific E3 ubiquitin ligase MDM2 (also known as HDM2) in the cytoplasm was augmented. These results strongly suggest that the increased interaction between p53 and MDM2, in the cytoplasm, impaired both the nuclear translocation and the activity of p53. This interaction between p53 and MDM2 was regulated by mitogen-activated protein kinase/extracellular signal-regulated kinase kinase extracellular signal-regulated kinase (MEK-ERK)-induced phosphorylation at Ser^166^ in the MDM2 protein. Interestingly, MEK-ERK phosphorylation of MDM2 was liver specific [38].

## 5. Conclusion

The results of our studies showed a novel HCV-induced pathway that activates DHCR24 in response to oxidative stress. Overexpression of DHCR24 by HCV contributed to the development of HCC during persistent HCV infections. Recently, we found that silencing of DHCR24 by siRNA suppresses HCV replication [39] and an inhibitor of DHCR24 (U18666A) had an anti-viral effect *in vivo*. Monoclonal antibodies to DHCR24 (2-152a) suppress HCV replication through the betaine GABA transporter-1 (BGT-1) [40]. Thus, DHCR24 is involved in HCV replication and pathogenicity. DHCR24 catalyzes the reduction of the delta-24 bond of the sterol intermediate and works further downstream of farnesyl pyrophosphate, and therefore does not influence geranylgeranylation. Our findings may indicate the possible existence of a regulatory pathway of HCV replication by cholesterol synthesis and trafficking through DHCR24 in addition to protein geranylgeranylation. DHCR24 deficiency reduces cholesterol levels and disorganizes cholesterol-rich detergent-resistant membrane domains (DRMs) in mouse brains. Additionally, the HCV replication complex has been detected in the DRM fraction. Therefore, a deficiency in DRM, induced by silencing of DHCR24, may suppress HCV replication. In addition, BGT-1 plays a role in tonicity regulation and hyper-osmolarity [41], and recent reports show that hyperosmotic shrinkage stimulates duck hepatitis B virus replication [42]. BGT-1 is involved in sodium and chloride coupled betaine uptake and betaine levels affect lipid distribution even to such an extent that low plasma betaine levels correlate with unfavorable lipid profiles [43]. Future study will clarify the regulatory role of DHCR24 and BGT-1 in HCV replication.

In conclusion, the results of our studies suggest that HCV infected cells may become anti-apoptotic and replicate efficiently to establish chronic infection through over-expression of DHCR24. Thus, the HCV-induced oxidative stress responsive protein DHCR24 may play a critical role in the pathogenesis of HCV persistent infections.
